# Identification of novel driver mutations of the discoidin domain receptor 2 (DDR2) gene in squamous cell lung cancer of Chinese patients

**DOI:** 10.1186/1471-2407-14-369

**Published:** 2014-05-24

**Authors:** Liyun Miao, Yongsheng Wang, Suhua Zhu, Minke Shi, Yan Li, Jingjing Ding, Jun Yang, Qing Ye, Hourong Cai, Deping Zhang, Hongbing liu, Yong Song

**Affiliations:** 1 Department of Respiratory Medicine, Nanjing Drum Tower Hospital Affiliated to Medical School of Nanjing University, Zhongshan Road 321#, Nanjing 210008, China; 2 Department of Respiratory Medicine, Jinling Hospital, Medical School of Nanjing University, East Zhongshan Road 305#, Nanjing 210002, Jiangsu Province China; 3 Department of Cardiothoracic Surgery, Nanjing Drum Tower Hospital Affiliated to Medical School of Nanjing University, Zhongshan Road 321#, Nanjing 210008, China; 4 Department of pathology, Nanjing Drum Tower Hospital Affiliated to Medical School of Nanjing University, Zhongshan Road 321#, Nanjing 210008, China

**Keywords:** Squamous cell lung cancer, DDR2 mutation, Proliferation, Invasion, E-cadherin

## Abstract

**Background:**

Although many of the recently approved genomically targeted therapies have improved outcomes for patients in non–small-cell lung cancer (NSCLC) with lung adenocarcinoma, little is known about the genomic alterations that drive lung squamous cell cancer (SCC) and development of effective targeted therapies in lung SCC is a promising area to be further investigated. Discoidin domain receptor 2 (DDR2), is a novel receptor tyrosine kinases that respond to several collagens and involved in tissue repair, primary and metastatic cancer progression.

**Methods:**

Expression of DDR2 mRNA was analyzed in 54 lung SCC tissues by qRT-PCR. Over-expression approaches were used to investigate the biological functions of DDR2 and its’ mutations in lung SCC cells. Conventional Sanger sequencing was used to investigate the mutations of DDR2 gene in 86 samples. The effect of DDR2 and its’ mutations on proliferation was evaluated by MTT and colony formation assays; cell migration and invasion was evaluated by trasnwell assays. Lung SCC cells stably transfected with pEGFP-DDR2 WT, pEGFP-DDR2-S131C or empty vector were injection into nude mice to study the effect of DDR2 and its’ mutation on tumorigenesis in *vivo*. Protein and mRNA expression levels of E-cadherin and MMP2 were determined by qRT-PCR and western blot analysis. Differences between groups were tested for significance using Student’s t-test (two-tailed).

**Results:**

In this study, we found that DDR2 mRNA levels were significantly decreased in 54 lung SCC tissues compared with normal lung tissues. Moreover, there were 3 novel DDR2 mutations (G531V, S131C, T681I) in 4 patients and provide the mutation rate of 4.6% in the 86 patients with lung SCC. The mutation of S131C in DDR2 could promote lung SCC cells proliferation, migration and invasion via inducing MMP-2, but reducing E-cadherin expression.

**Conclusions:**

These data indicated that the novel DDR2 mutation may contribute to the development and progression of lung SCC and this effect may be associated with increased proliferation and invasiveness, at least in part, via regulating E-cadherin expression.

## Background

Lung cancer is the leading cause of cancer death worldwide, and Non-small cell lung cancer (NSCLC) that including adenocarcinoma and squamous cell carcinoma, is the predominant form of lung cancer [1]. Because of the limited benefits provided by surgery, chemotherapy, and radiation, the improvement in prognosis and survival of patients with lung cancer in the past 20 years is still unfavorable [2]. Recently, although significant advances have achieved in the chemotherapy and radiation therapy for advanced disease patients with NSCLC; however, most patients will eventually develop resistance. Therefore, there is a need for better understanding of the genetic abnormalities in NSCLC cancers to identify and develop novel and effective targeted therapies.

To date, analysis of individual patients’ genetic makeup is becoming more and more important in guiding the development of novel treatments. A striking example of this is the development of small-molecule inhibitors of the epidermal growth factor receptor (EGFR) tyrosine kinase therapies, which resulted in a great deal of progress in the targeted treatment of patients with NSCLC [3-6]. Somatic mutations in the EGFR gene play key roles in determining the sensitivity of NSCLC patients treated with EGFR inhibitor drugs; however, most of the patients who respond to EGFR kinase inhibitors are the adenocarcinoma subtype of NSCLC [7]. In contrast, patients with the lung squamous cell cancer (SCC) which accounts for about 25% of NSCLC very rarely respond to these agents; few advances have been made in the treatment of this type of NSCLC [8]. In addition to EGFR, many other promising therapeutic targets including EML4-ALK, MET and KRAS have been identified and drugs directed against these proteins are being tested in clinical trials [9-11]. However, it appears that these drugs are also likely limited to lung adenocarcinomas. Given the burden of disease from lung SCC, identifying new therapeutic targets of mutated kinases is essential for lung SCCs.

DDR2, a receptor tyrosine kinase that binds collagen I and III as its endogenous ligand, is known to increase expression of matrix metalloproteinases and has been previously shown to promote cell proliferation, migration and metastasis by regulating epithelial–mesenchymal transition [12-15]. The altered expression patterns of DDR2 mRNA expression have been reported in multiple types of human cancer, including NSCLC [16,17]. Moreover, DDR2 mutations have been noted in several cancer specimens including in NSCLC (R105S, H136H and N456S) [18,19]. However, these reports have not been confirmed in independent samples and whether there are novel mutations in Chinese population should be investigated.

In this study, the mRNA levels and mutation status of DDR2 at the discoidin and kinase domains in lung SCC was investigated. We found three novel somatic mutations in the DDR2 at a frequency of 4.6% ( n = 4) in a sample set of 86 lung SCC samples. We also show that DDR2 mutations are oncogenic via promoting cells proliferation, migration and invasion by exogenous overexpression in lung SCC cells. Furthermore, DDR2 mutation could induce Epithelial-to-Mesenchymal Transition in lung SCC cells by downregulating E-cadherin expression. These data indicated that the novel DDR2 mRNA mutation may contribute to the development and progression of lung SCC and this effect may be associated with increased proliferation and invasiveness, at least in part, via regulating E-cadherin expression.

## Methods

### Tissue samples

Lung SCC and normal lung tissues were obtained from patients who underwent primary surgical resection of NSCLC with informed consent at Nanjing Drum Tower Hospital Affiliated to Medical School of Nanjing University between 2008 and 2011. No local or systemic treatment had been conducted in these patients before the operation. All these tissue samples were immediately snap-frozen in liquid nitrogen and stored at -80°C until total RNA was extracted. The study was approved by the Research Ethics Committee of Nanjing Drum Tower Hospital (Nanjing, Jiangsu, PR China). Informed consent was obtained from all patients.

### Cell lines and culture conditions

NSCLC squamous carcinomas cell lines (SK-MES-1, NCI-H1703), a normal human bronchial epithelial cell line (HBE) were purchased from the Institute of Biochemistry and Cell Biology of the Chinese Academy of Sciences (Shanghai, China). All cells were cultured in RPMI 1640 (GIBCO-BRL) medium supplemented with 10% fetal bovine serum (10% FBS), 100 U/ml penicillin, and 100 mg/ml streptomycin (invitrogen) in humidified air at 37°C with 5% CO2. Cells were grown on 250 ng/ml type I collagen (BD Biosciences, San Diego, CA) for all relative experiments.

### RNA extraction and qRT-PCR analyses

Total RNA was isolated with TRIzol reagent (Invitrogen, Carlsbad, CA, USA) according to the manufacturer’s protocol. For analysis of DDR2, E-cadherin, N-cadherin, MMP-2 and MMP-9 mRNA expression, 500 ng total RNA was reverse transcribed in a final volume of 10 μl using random primers under standard conditions using the PrimeScript RT reagent Kit and SYBR Premix Ex Taq (TaKaRa, Dalian, China) according to the manufacturer’s instructions. GAPDH gene was used as an internal control. The primers were designed as follows: DDR2, forword primer: 5′-CCACTATGCAGAGGCTGACA-3′ and reverse: 5′-CAGAGATGAACCTCCCCAAA-3′; MMP-2, forword primer: 5′ CGTCTGTCCCAGGATGACATC 3′, reverse primer: 5′TGTCAGGAGAGGCCCCATAG 3′; MMP-9, forword primer: 5′ TGGGCAGATTCCAAACCTTT 3′, reverse primer: 5′ TCTTCCGAGTAGTTTTGGATCCA 3′; E-cadherin, forword primer: 5′ TCCCATCAGCTGCCCAGAAA 3′, reverse primer:5′ TGACTCCTGTGTTCCTGTTA 3′GAPDH, forword primer: 5′ GGGAGCCAAAAGGGTCAT 3′, reverse primer: 5′ GAGTCCTTCCACG ATACCAA 3′. The relative levels of mRNA expression were calculated based on the difference between amplification of target genes and GAPDH mRNA using the 2^-ΔΔ^ct method. All experiments were performed three times with three technical replicates.

### DDR2 sequencing

DDR2 was sequenced from DNA obtained from lung SCC patient samples by conventional Sanger sequencing. In the discovery set, 86 patient samples were used for sequencing DDR2 gene mutation. All mutations were confirmed as somatic. Mutations were identified using an automated mutation caller and then verified manually with comparison made to the matched normal sequence in the case of all primary tumor samples.

### Plasmid constructs

To generate a DDR2 and its mutated transcript expression vector, the entire sequence of human DDR2 and mutatedDDR2 was synthesized and subcloned into pEGFP-N1 vector with incorporate external *NheI* and *BamHI* sites, respectively (Invitrogen, Shanghai, China).

### Transfection of lung SCC cells

All plasmid vectors for transfection were extracted by DNA Midiprep or Midiprep kit (Qiagen, Hilden, Germany). Lung SCC cells cultured on six-well plate were transfected with the pEGFP-DDR2, pEGFP-DDR2-S131C, pEGFP-DDR2-T681I or empty vector using Lipofectamine2000 (Invitrogen, Shanghai, China) according to the manufacturer’s instructions. Cells were harvested after 48 hours for qRT-PCR and western blot analyses.

### Cell proliferation assays

Cell proliferation assay was performed with MTT kit (Sigma, St. Louis, Mo) according to the manufacturer’s instruction. Cells were placed into 6-well plate and maintained in media containing 10% FBS for 2 weeks for colony formation assay. Colonies were fixed with methanol and stained with 0.1% crystal violet (Sigma, St. Louis, Mo). Visible colonies were manually counted.

### Cell migration and invasion assays

For the migration assays, 24 hours after transfection, 3 × 10^4^ cells in serum-free media were placed into the upper chamber of an insert (8-μm pore size, millepore). For the invasion assays, 1 × 10^5^ cells in serum-free media were placed into the upper chamber of an insert coated with Matrigel (BD, San Diego, CA. Media containing 10% FBS were added to the lower chamber. After 24 hours of incubation, the cells remaining on the upper membrane were removed with cotton wool, whereas the cells that had migrated or invaded through the membrane were stained with methanol and 0.1% crystal violet, imaged, and counted using an IX71 inverted microscope (Olympus, Tokyo, Japan). Experiments were independently repeated three times.

### Western blotting assay

Cells were lysed using mammalian protein extraction reagent RIPA (Beyotime) supplemented with protease inhibitors cocktail (Roche) and PMSF (Roche). Protein concentration was measured with the Bio-Rad protein assay kit. 40 μg protein extractions were separated by 10% SDS-polyacrylamide gel electrophoresis (SDS-PAGE), then transferred to 0.22 μm NC membranes (Sigma) and incubated with specific antibodies. Autoradiograms were quantified by densitometry (Quantity One software; Bio-Rad). GAPDH was used as control. GAPDH antibody was purchased from sigma; Collagen Iand DDR2 antibody were purchased from Abcam; E-cadherin antibody was purchased from BD (San Diego, CA); MMP-2 antibody was purchased from CST.

### Tumor formation assay in a nude mouse model

Four weeks old nude mice were used for the tumor formation assay. All of the mice were BALB/c background. The animal care and experimental procedures were approved by the Model Animal Research Center of Jingling Hospital and conducted according to Institutional Animal Care and User guidelines. H1703 cells stably transfected with pEGFP-DDR2, pEGFP-DDR2-S131C or empty vector were resuspended at a concentration of 2 × 107 cells/ml. Each mouse was injected on the right side of the posterior flank with 2 × 106 suspended cells. Tumor growth was measured by calipers every 3 days. The tumors were removed from all of the animals after 15 days, and the subcutaneous growth of each tumor was examined. The tumor volumes were calculated using the equation V = 0.5 × D × d2 (V, volume; D, longitudinal diameter; d, latitudinal diameter). All of the surgeries were performed under sodium pentobarbital anesthesia, and all efforts were made to minimize suffering.

### Statistical analysis

Student’s t-test (two-tailed), One-way ANOVA and Mann–Whitney test were performed to analyze the data using SPSS 16.0 software. P values less than 0.05 were considered statistically significant.

## Results

### Expression of DDR2 mRNA is down-regulated in lung SCC

**Figure 1:**
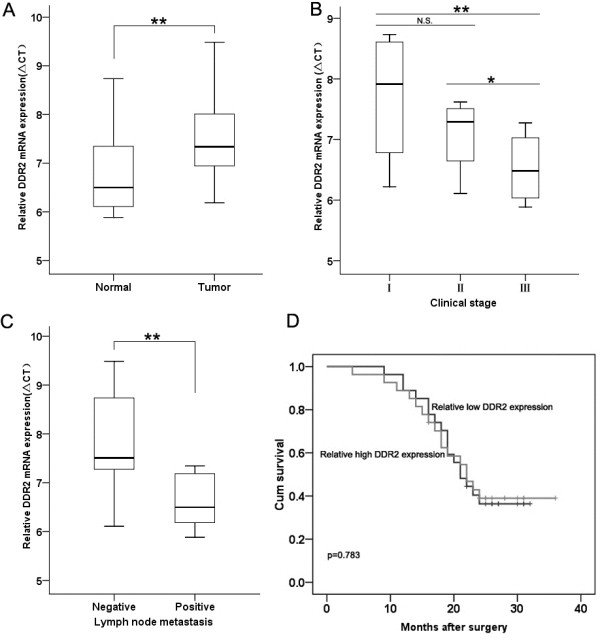
**Relative DDR2 expression in lung SCC tissues and its clinical significance. (A)** qRT–PCR analysis of the relative DDR2 expression in lung SCC tissues (n = 54) and in paired adjacent normal tissues (n = 54). DDR2 expression was normalized to GAPDH expression. The data are presented as a fold-change in the tumor tissue relative to the normal tissue. **(B)** DDR2 expression was relatively higher in patients with lymph node metastasis. **(C)** DDR2 expression was relatively higher in patients with an advanced clinical stage than those with an early clinical stage. **(D)** There was no significantly difference in survival times between patients with high DDR2 expression and those with low DDR2 expression levels *P < 0.05, **P < 0.01.

The expression of DDR2 was detected in 54 lung SCC samples and normal tissues by qRT-PCR, and normalized to GAPDH. The level of DDR2 mRNA was significantly decreased in cancerous tissues (median ratio of 1.76-fold, p < 0.01) compared with corresponding normal tissues (Figure 1A). Furthermore, correlation analysis of DDR2 expression with clinical pathological features of lung SCC patients showed that DDR2 expression was relatively higher in lung SCC patients with advanced stage (*P* = 0.006) and lymph node metastasis (*P* = 0.009) (Figure 1B and C). However, DDR2 expression was not correlated with patient age, gender or other clinicopathological features (data not shown).

Kaplan-Meier survival analysis was performed to further evaluate the correlation between DDR2 expression and lung SCC patient prognosis. According to the median ratio of relative DDR2 expression (1.76) in tumor tissues, the 56 NSCLC patients were classified into two groups: High-DDR2 group (n = 27, DDR2 expression ratio ≥ median ratio) and Low-DDR2 group (n = 27, DDR2 expression ratio ≤ median ratio). The Kaplan-Meier survival curve showed that there was no significantly difference in survival times between patients with high DDR2 expression and those with low DDR2 expression levels (Figure 1D).

### DDR2 is mutated in lung SCC

**Figure 2:**
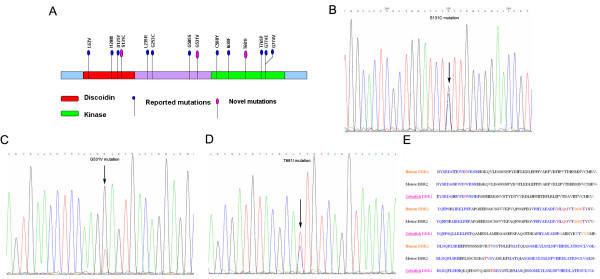
**Detection of DDR2 gene mutation in lung SCC tissues.** Conventional Sanger sequencing was performed to analysis the DRR2 gene mutation. **(A)** In addition to reported DDR2 mutations, three novel DDR2 muatations were found in chinese lung SCC patients. **(B, C and D)** The S131C mutation was identified in the exon5, G531V and T681I mutations were found in exon13 and exon15, respectively. **(E)** Analyse of amino acid conservation of DDR2 among human, mouse, and zebrafish homologs.

We performed Sanger sequencing of DDR2 gene in an set of 86 primary lung SCC samples and identified four synonymous mutations in 7 samples and three novel recurrent somatic mutations (G531V, S131C, T681I) in 4 samples in the tyrosine kinase genes: DDR2, resulting in an overall frequency of 4.6% in 86 total primary lung SCC samples. Mutations were found both in the kinase domain and in other regions of the protein sequence (Figure 2A). The S131C mutation was identified in the exon5, G531V and T681I mutations were found in exon13 and exon15, respectively (Figure 2B, C and D). The majority of the mutations resided in regions of high degrees of amino acid conservation, compared with the mouse, and zebrafish homologs of DDR2 (Figure 2E). A query of the limited clinical information accompanying the sequenced samples did not identify any significant correlation of DDR2 mutation status with age, sex, or smoking status of the patients (Additional file 1: Table S1).

### DDR2 S131C mutation is oncogenic and promotes lung SCC cells proliferation in *vitro*

**Figure 3:**
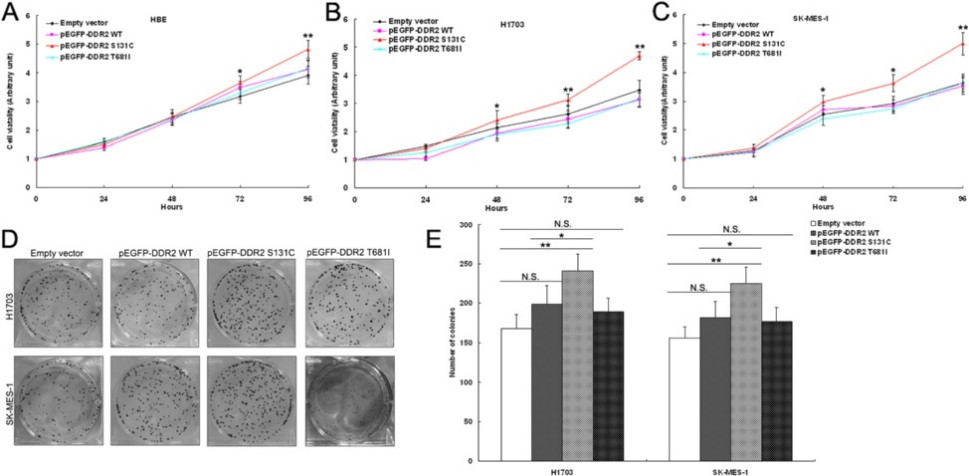
**The effect of DDR2 mutation on lung SCC cells proliferation in vitro.** HBE, H1703 and SK-MES-1 cells were transfected with pEGFP-DDR2, pEGFP-DDR2-S131C, pEGFP-DDR2-T681I or empty vector. **(A, B, C)** An MTT assay was performed to determine the proliferation of pEGFP-DDR2, pEGFP-DDR2-S131C, pEGFP-DDR2-T681I or empty vector-transfected HBE, H1703 and SK-MES-1 cells. **(D,E)** A colony-forming growth assay was performed to determine the proliferation of pEGFP-DDR2, pEGFP-DDR2-S131C, pEGFP-DDR2-T681I or empty vector-transfected HBE, H1703 and SK-MES-1 cells. The colonies were counted and captured.

DDR2 mutations have been found to be associated with lung SCC cells growth and dasatinib sensitivity. Therefore, to investigate the potential biological function of these novel DDR2 mutations in lung SCC cells, we constructed the DDR2 wild type, S131C and T681I mutated DDR2 expression plasmid vector (pEGFP-DDR2, pEGFP-DDR2- S131C, pEGFP-DDR2- T681I). Furthermore, MTT assay revealed that cell growth was significantly increased in HBE, H1703 and SK-MES-1 cells transfected with pEGFP-DDR2-S131C compared with cells transfected with empty vector, wildtype pEGFP-DDR2 or pEGFP-DDR2-T681I vector (Figure 3A, B and C). Similarly, the results of colony-formation assay also showed that clonogenic survival was increased following transfection of pEGFP-DDR2-S131C in HBE, H1703 and SK-MES-1 cells (Figure 3D).

### Effect of DDR2 S131C mutation on lung SCC cells migration and invasion

**Figure 4:**
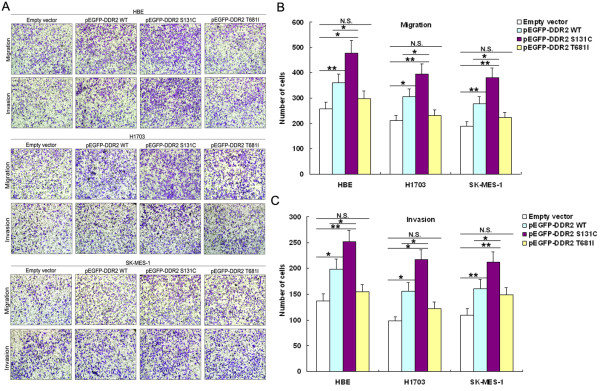
**The effect of DDR2 mutation on lung SCC cells migration and invasion in vitro.** HBE, H1703 and SK-MES-1 cells were transfected with pEGFP-DDR2, pEGFP-DDR2-S131C, pEGFP-DDR2-T681I or empty vector. **(A, B, C)** Transwell assays were used to investigate the changes in migratory and invasive abilities of cells. *P < 0.05 and **P < 0.01.

Recently, DDR2 was reported to be critical for breast cancer invasion and migration in vitro and for metastasis in vivo via sustaining SNAIL1 stability and activity to promote tumor cells migration and invasion through collagen-I-enriched tumour-associated matrices [15]. To investigate whether DDR2 mutation could have a direct functional effect in facilitating lung SCC cell migration and invasion, we evaluated cancer cell invasion through matrigel and migration through wound healing and transwell assays. As shown in Figure 4A, overexpression of DDR2 S131C could enhance the ability of migration and invasion in HBE cells when compared with cells treated with pEGFP-DDR2 wildtype vector. Similarly, migration and invasion of H1703 and SK-MES-1 cells was also increased following transfection of pEGFP-DDR2-S131C compared with cells transfected with empty vector, wildtype pEGFP-DDR2 or pEGFP-DDR2-T681I vector (Figure 4B and C). These data indicated that DDR2 S131C mutation can promote the migratory and invasive phenotype of lung SCC cells.

### DDR2 S131C mutation promotes lung SCC cells growth in *vivo*

**Figure 5:**
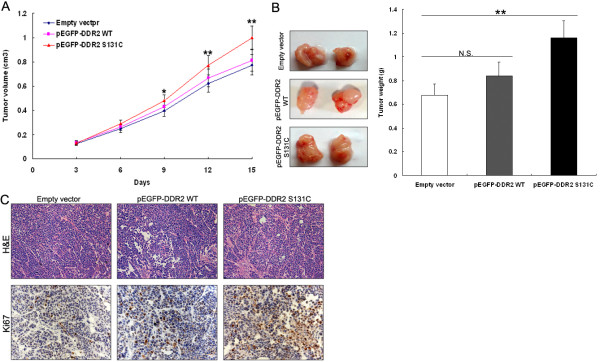
**DDR2 S131C mutation promotes lung SCC cells growth in *vivo*. (A)** Tumor growth curve. H1703 cells were stablely transfected with pEGFP-DDR2, pEGFP-DDR2-S131C or empty vector and then injected into nude mice. The error bars represent the standard deviation (S.D). **(B)** The tumor weight was represented as means of tumor weights ± s.d. **(C)** Representative images (×200) of HE staining and immunohistochemistry of the proliferation index Ki67.

To further provide in vivo evidence for the oncogenic role of DDR2 S131C mutation in lung SCC, we used a xenograft mouse model. BALB/c mice were subcutaneously injected with H1703 cells transfected with pEGFP-DDR2, pEGFP-DDR2-S131C or empty vector randomly. Three days after injection, all of them developed detectable tumors. Compared to the control treatment, DDR2-S131C overexpression treatment dramatically increased tumor growth, which was demonstrated by significantly increased tumor size and weight (Figure 5A and B). Thus, DDR2-S131C overexpression promotes the growth of established lung SCC xenografts. In addition, the HE staining showed the typical characteristics of tumor cells, and the proliferation index Ki67 determined by immunohistochemical staining significantly upregulated in the pEGFP-DDR2-S131C transfected tumors (Figure 5C).

### DDR2 mutation induced lung cells proliferation and invasion partly via regulating E-cadherin expression

Firstly, we investigated the total DDR2 protein levels of H1703 cells after transfection of wildtype or mutated DDR2 and the results that there was no difference in wildtype or mutated DDR2 transfected H1703 cells. Furthermore, to investigate whether these mutations affect collagen binding, we detected the collagen Iprotein level in wildtype or mutated DDR2 transfected H1703 cells;however, there was no significantly difference. These data indicated that the observed phenotypes is not due to differences in protein expression levels or collagenI binding, which may be due to receptor phosphotyrosine levels upon acquisition of mutations.

**Figure 6:**
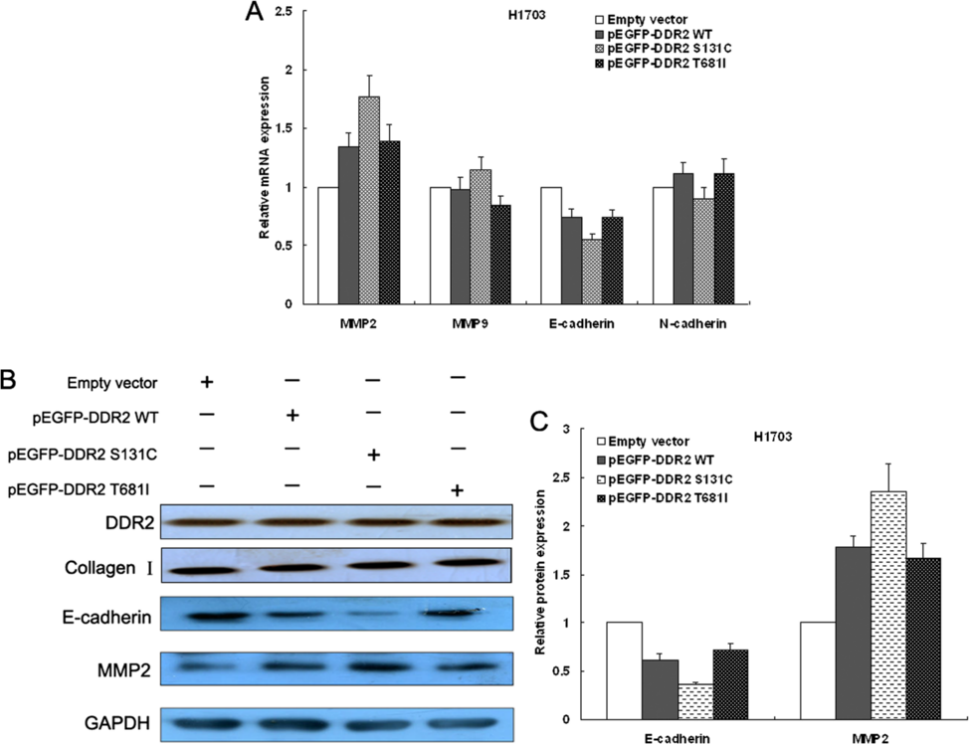
**E-cadherin is key downstream mediator of DDR2 S131C.** H1703 cells were transfected with pEGFP-DDR2, pEGFP-DDR2-S131C, pEGFP-DDR2-T681I or empty vector, respectively. **(A)** QRT-PCR analysis was performed to determine E-cadherin, N-cadherin, MMP2 and MMP9 expression in H1703 cells. **(B, C)** E-cadherin and MMP2 protein expression in H1703 cells was determined by western blot analysis. The results are from 3 independent experiments. GAPDH protein expression was used as an internal control. * P < 0.05; ** P < 0.01.

Epithelial-to-mesenchymal transition (EMT), a fundamental biological process in embryonic development, has been found to be involved in tissue homeostasis, wound healing, tumor invasion and metastasis [20]. Recent studies show that transforming Growth Factor-beta1 (TGF-β1) could promote increased expression of type I collagen and DDR2 and induce EMT, while knockdown of DDR2 expression with siRNA inhibits EMT directly induced by type I collagen [14,21]. Therefore, we investigated whether the mechanism whereby DDR2 mutation could promote EMT process in lung SCC cells. The results of qRT-PCR showed that DDR2 ovexpression could induce the MMP-2 mRNA expression and decrease E-cadherin mRNA expression, while transfection of pEGFP-DDR2-S131C could induce more significantly changes in E-cadherin and MMP-2 mRNA expression (Figure 6A). Moreover, western blot analysis also showed the same results (Figure 6B and C). These data indicated that DDR2 mutation may infuence lung SCC cells proliferation, migration and invasion via partly promoting the epithelial-mesenchymal transition.

## Discussion

Despite the deployment of molecularly targeted agents leading to great advances in the treatment of lung adenocarcinoma and improvements in patient outcomes, little is currently known about the targetable genetic abnormalities underlying lung SCC [22,23]. In addition to TP53 mutations, lung SCC have been shown to harbor amplifications of SOX2 and EGFR variant III mutations as well as DDR2 mutations [18,24,25]. In the present study, we found that DDR2 mRNA expression is significantly downregulated in lung SCC tissues when compared with normal lung tissue. Moreover, 3 novel mutations in exon5, 13 and 15 of DDR2 gene in a screen of 86 lung SCC samples were identified, yielding an overall mutation rate of 4.6% in all samples, which indicated that there is no significant difference of DDR2 mutation rate in Chinese, Europe and American patients. However, DDR2 mutation does not exist concentrated area and missense mutation are more slightly common in the extracellular domain and kinase domain. DDR2 have previously been reported to be involved in various human diseases, including cancers [26-28]. Although the sample size was not large, the novel DDR2 mutations in lung SCC suggest that DDR2 mutations could contribute to the pathogenesis of lung SCC.

The mechanism by which DDR2 and its mutations may contribute to oncogenesis in lung SCC is not well known; however, given its role in transmitting signals from the ECM, it is likely that DDR2 could act as regulators of cell proliferation, migration and subsequent tumor cells metastasis. Activated DDR2 can induce the expression of MMP-1, MMP-2 and MMP-13, and stimulation of DDR2 could promote fibroblast migration and proliferation. In addition, it is conceivable that altered expression of DDRs triggers abnormal activity, ultimately leading to enhanced proliferation and oncogenesis as well as EGFR [29-31]. In this study, DDR2 wildtype overexpression vector and two DDR2 mutations vector (pEGFP-DDR2-S131C, pEGFP-DDR2-T681) were constructed and transfected into HBE and lung SCC cells to explore the potential biological function and underlying molecular mechanism of DDR2 and its mutations in lung SCC development. The results showed that ectopic expression of mutant forms of DDR2 could function as an oncogene in either context. Further investigation indicated that enhanced DDR2 and its S131C mutation could promote HBE and lung SCC cells proliferation, migration and invasion partly via promoting EMT through regulating MMP-2 and E-cadherin expression. These data indicated that mutations in discodin region may contribute to more biologically function than mutations in kinase region.

EMT is firstly recognized as a central differentiation process allowing the remodeling of tissues during early embryogenic and is implicated in the promotion of tumor invasion and metastasis [20]. EMT can be initiated by external signals originating from outside the cell, such as transforming growth factor (TGF)-b, hepatocyte growth factor (HGF), epidermal growth factor (EGF), and fibroblast growth factor (FGF) [32,33]. Furthermore, it has been proposed and supported by numerous publications that EMT process would be a potent mechanism that enhances the detachment of cancer cells from primary tumors. One characteristic of cells that undergone EMT is the loss of E-cadherin expression, and decreased E-cadherin expression has been reported to be associated with poor clinical outcome in NSCLC [34,35]. Therefore, EMT-inducing pathways may be good candidates for intervention in the treatment of cancer, and it is important to understand the molecular mechanisms that drive EMT for the prevention of metastasis. In this study, we showed that DDR2 and its mutation is an effective regulatory factor promoting EMT in lung SCC cells.

## Conclusions

In conclusion, the DDR2 expression pattern and mutations in lung SCCs patients was observed in this study. However, we did not evaluate the effects of expression of mutated DDR2 in an large enough sample size to detect a statistically significant difference in the rates of DDR2 mutation, nor did we complete an assessment of function of all identified DDR2 mutants in lung SCC cells. Finally, this study provides evidence that novel DDR2 mutations in lung SCC, and at least one of which is functionally significant adding to the knowledge of the genetic landscape of SCCs. We hope our data may stimulate the initiation of larger clinical trials of testing of lung SCC patients for DDR2 mutations leading to a more effective treatment for this deadly disease.

## Competing interests

The authors declare that they have no competing interests.

## Authors’ contributions

MLY, WYS and SY were involved in the conception and design of the study. ZSH, SMK, ZPP and LY was involved in the provision of study material and patients. DJJ, YJ, YQ, CHR and LHB performed the data analysis and interpretation. MLY wrote the manuscript. SY approved the final version. All authors read and approved the final manuscript.

## Pre-publication history

The pre-publication history for this paper can be accessed here:

http://www.biomedcentral.com/1471-2407/14/369/prepub

## Supplementary Material

Additional file 1: Table S1 Clinicopathological characteristics and DDR2 mutations of 86 patient samples of lung SCC. Click here for file
